# Peer Review of “Chaotic and Stochastic Components in an Influenza Surveillance Series: Nonlinear Dynamics and Predictive Modeling Study”

**DOI:** 10.2196/101473

**Published:** 2026-06-05

**Authors:** Sokaina El Khamlichi

**Affiliations:** 1 Ibn Zohr University, Agadir, Morocco

**Keywords:** epidemiology, epidemiologic methods, epidemiological monitoring, chaos theory, topological data analysis, autoregressive conditional heteroskedasticity

*This is a peer-review report for “Chaotic and Stochastic Components in an Influenza Surveillance Series: Nonlinear Dynamics and Predictive Modeling Study.”*

## Round 1 Review

### General Comments

This paper [1] presents an interesting application of chaos theory, nonlinear time series analysis, and topological machine learning to influenza epidemiological data. The methodology is strong and promising. However, the manuscript requires substantial improvements to enhance clarity and accessibility.

### Specific Comments

#### Major Comments

1. Abstract: Currently very dense, heavy with jargon, and difficult for nonspecialists to follow. Please simplify sentences and clearly highlight the main objective, methodology, key findings, and implications.

2. Introduction: Starts too technically. Begin with a motivation, for instance, “Why chaos theory in epidemiology?” and then gradually introduce deterministic vs stochastic chaos. Also, state the study’s aim more explicitly.

3. Methods: The section is lengthy, with equations embedded in the text. Consider dividing into subsections for readability. Moving some theoretical background to an appendix would also improve flow. Please add a figure summarizing the main steps of the study.

4. Results: The analysis is rich but scattered. Figures and tables are described without sufficient interpretation. For each, explicitly state the following: What do we see? What does it mean? Why does it matter?

5. Discussion: A separate Discussion section would strengthen the paper. You can structure it as follows: evidence of stochastic chaos in influenza, implications for epidemiological modeling and prediction, and comparison with previous applications of chaos theory.

6. Conclusion: Currently too technical and lacks a clear take-home message. Please make it concise, accessible, and impact-driven.

#### Minor Comments

7. Language and style: Reduce the frequent repetition of the phrase “stochastic chaotic.” Simplify overly long sentences for better readability.

## Round 2 Review

### General Comments

This paper presents an interesting and technically rich application of chaos theory, nonlinear time series analysis, and topological machine learning to influenza epidemiological data. The methodology is strong, and the topic is relevant for epidemiological modeling and risk analysis.

The authors have made some efforts to improve the manuscript following the first round of review, particularly in terms of organization and presentation. However, while progress has been made, several of the initial comments remain only partially addressed, and further revisions are required.

### Specific Comments

#### Major Comments

1. Although some improvements in clarity are noticeable, the manuscript remains dense and difficult to follow, particularly in the Abstract, Methods, and Results sections.

2. The Abstract remains too long and should be further simplified and condensed.

3. The structure of the Methods section could still be improved. The addition of a clear workflow diagram summarizing the methodology would greatly enhance readability.

4. The Results section continues to lack sufficient interpretation of figures and tables. The authors should more clearly explain what each result shows and why it is important.

## Round 3 Review

### General Comments

The manuscript presents a technically solid and original study combining chaos theory, nonlinear time series analysis, and machine learning methods for influenza surveillance data. The authors have addressed the main concerns raised during the review process, particularly regarding the organization of the methodology and the interpretation of the results.

The paper provides a substantial amount of analysis, and the conclusions are generally supported by the reported findings. While some sections remain relatively dense and could still benefit from minor language polishing and simplification, the manuscript is now sufficiently clear and coherent for publication.
